# A New Approach for Impedance Tracking of Piezoelectric Vibration Energy Harvesters Based on a Zeta Converter

**DOI:** 10.3390/s20205862

**Published:** 2020-10-16

**Authors:** Antonino Quattrocchi, Roberto Montanini, Salvatore De Caro, Saverio Panarello, Tommaso Scimone, Salvatore Foti, Antonio Testa

**Affiliations:** Department of Engineering, University of Messina, 98166 Messina, Italy; roberto.montanini@unime.it (R.M.); salvatore.decaro@unime.it (S.D.C.); saverio.panarello@unime.it (S.P.); tommaso.scimone@unime.it (T.S.); salvatore.foti@unime.it (S.F.); antonio.testa@unime.it (A.T.)

**Keywords:** piezoelectric generators, piezoceramic patches, energy harvesting, DC–DC power converters, energy conversion efficiency, impedance matching

## Abstract

Piezoelectric energy harvesters (PEHs) are a reduced, but fundamental, source of power for embedded, remote, and no-grid connected electrical systems. Some key limits, such as low power density, poor conversion efficiency, high internal impedance, and AC output, can be partially overcome by matching their internal electrical impedance to that of the applied resistance load. However, the applied resistance load can vary significantly in time, since it depends on the vibration frequency and the working temperature. Hence, a real-time tracking of the applied impedance load should be done to always harvest the maximum energy from the PEH. This paper faces the above problem by presenting an active control able to track and follow in time the optimal working point of a PEH. It exploits a non-conventional AC–DC converter, which integrates a single-stage DC–DC Zeta converter and a full-bridge active rectifier, controlled by a dedicated algorithm based on pulse-width modulation (PWM) with maximum power point tracking (MPPT). A prototype of the proposed converter, based on discrete components, was created and experimentally tested by applying a sudden variation of the resistance load, aimed to emulate a change in the excitation frequency from 30 to 70 Hz and a change in the operating temperature from 25 to 50 °C. Results showed the effectiveness of the proposed approach, which allowed to match the optimal load after 0.38 s for a ΔR of 47 kΩ and after 0.15 s for a ΔR of 18 kΩ.

## 1. Introduction

Energy harvesting deals with the regeneration of surplus environmental energy and is attracting an increasing interest, powered by the race towards the reduction of size and weight of electrical and electronic devices. Today, energy harvesters are only starting to be considered as a viable alternative to inexpensively supply different types of mobile, remote-controlled, or completely autonomous devices, but, in a near future, one or more energy harvesters could be embedded into most consumer electronic devices [1,2,3]. Electric energy can be obtained from waste energy of a different nature, such as mechanical vibrations, electromagnetic fields, light, heat, and pressure. Among them, the mechanical energy generated by residual vibrations is widely available in home, industrial, and vehicular environments, making the development of vibrational energy harvesters one of the most promising research fields, with the goal of turning wireless, wearable, and portable electronic devices into self-supplied systems [4].

Energy harvesters based on electromagnetic, piezoelectric, electrostatic, or electrochemical principles have been developed in the last few years to convert vibrations into electric power, which can be instantly used or stored. Among them, much attention is now being paid to piezoelectric devices, because the piezoelectric effect, based on the ability of some crystals to generate an electromotive force when subjected to a mechanical stress, makes it possible to recover residual mechanical energy from many sources, such as human motion, machine vibrations, acoustic noises, and thermal waste [5,6,7,8,9,10]. These piezoelectric devices can be used in several applications, taking advantage of their ability to work as either sensors or actuators [11,12]. However, the wide distribution of piezoelectric energy harvesters (PEHs) is currently burdened by some key limits, which include low power density, poor conversion efficiency when they do not work in resonance, high internal impedance, and AC output. Piezoelectric crystals or powders feature a power density of only a few µW/mm^2^, while the electrical power generated by a single piezoelectric device is quite small, ranging from some µW to some tens of mW. Hence, in order to fulfill the power requirements of standalone systems, such as GPS receivers, wearable electronic devices, or wireless sensors, practical piezoelectric harvesters are typically made by assembling multiple elementary devices. Piezoelectric generators that recover energy from vibrations are often based on mechanical resonators, which reach an acceptable efficiency only when working at resonance [13].

Furthermore, the applied electrical load plays an important role in determining the maximum output power that can be extracted from the PEH. According to the theorem of maximum power transfer, and in order to maximize the generated power, it should be very close to the internal impedance of the PEH. However, this optimal resistance load is also a function of the vibration frequency [14] and it also depends on the working temperature [15]. Hence, changes in the excitation vibration or in the environmental conditions leading to variation of the optimal resistance load should be appropriately faced by the PEH power converter in order to harvest the maximum energy.

**Figure 1:**
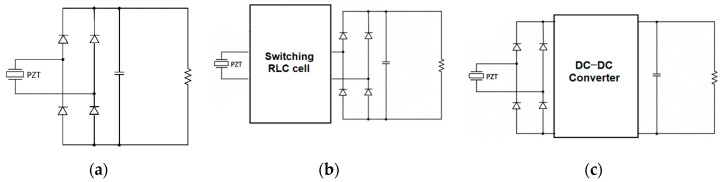
Conditioning circuits for piezoelectric energy harvesting based on (**a**) a full-wave diode rectifier, (**b**) a resonant rectifier, and (**c**) an active rectifier.

The main scope of the power converter is to turn the PEH AC output current into a DC one suitable for supplying electronic devices or batteries. As shown in Figure 1a, the easiest way for connecting a piezoelectric device to a DC load is to use a full-wave diode bridge. However, while diode rectifiers are simple and inexpensive, they are burdened by a quite low efficiency and they do not allow impedance tuning.

Actively controlled switching converters may be used to improve the conversion efficiency, but they involve higher costs and power self-consumption, thus a trade-off must be found between cost and energy yield. As shown in Figure 1b, resonant rectifiers have been proposed which exploit switching resistor–inductor–capacitor (RLC) circuits to optimally transfer power from a piezoelectric device to a diode rectifier. Active power devices are driven through self-synchronizing techniques, which do not need external control circuits, thus reducing the self-consumption [16,17,18,19]. A more effective approach relies on the exploitation of an active rectifier, which is composed of the cascade connection of a diode bridge and a suitable DC–DC converter, as shown in Figure 1c.

The DC–DC converter is tasked to regulate the DC output voltage and to draw the maximum possible power from the piezoelectric device for a specific electrical load [20,21,22,23,24,25]. The maximum power working point is obtained by controlling the output DC current in order to obtain an average converter input impedance close to that leading to the maximum power transfer. Such an approach, on the one hand, minimizes the number of sensors and the circuit self-consumption, but, on the other hand, it requires the knowledge of the optimal input resistance, which is not constant, being a function of the operating conditions. Buck, buck–boost, and flyback DC–DC converter topologies have been used to realize active rectifiers for piezoelectric generators, all featuring an inductive power transfer. Srinivasan et al. [26] used the principle of the buck–boost converter to achieve a bridgeless configuration for impedance matching based on single-stage direct AC–DC conversion. The results demonstrated that the harvested power is improved by the factors of 1.4 and 3.2 for single-input and multiple-input configurations, respectively, as compared to the power harvested using a dual-output rectifier. An alternative way to match the maximum power point can be provided by a step-down piezoelectric transformer, which can be integrated in a cantilever structure, connected to a bidirectional half-bridge converter [27].

All the above-mentioned schemes provide hardware configurations able to perform impedance matching, but they do not allow impedance tracking. Hence, if the electrical load applied to the PEH changes with time, they are no longer able to guarantee operation at the optimum working point.

In this paper, a new approach based on an active control is proposed to overcome this limitation, ensuring that maximum harvested energy is always extracted by the PEH as the applied electrical load undergoes variations due to changes in the excitation frequency or in the working temperature. The proposed hardware employs a non-conventional AC–DC converter that integrates a single-stage DC–DC Zeta converter and a full-bridge active rectifier. The implemented active control strategy is based on pulse-width modulation (PWM) coupled with maximum power point tracking (MPPT). The study presents results related to the effectiveness of the power optimization strategy; the operation of the proposed energy harvesting converter was evaluated by forcing a sudden variation of the applied resistance load. A preliminary characterization of the effect produced on the resistance load by a change in the working frequency (from 30 to 70 Hz) and in the operating temperature (from 25 to 50 °C) was carried out. These data were then used to emulate the variation of the load in practical situations.

## 2. PEH Electro-Mechanical Characterization

### 2.1. Cantilever-Based Piezoelectric Generator

**Figure 2:**
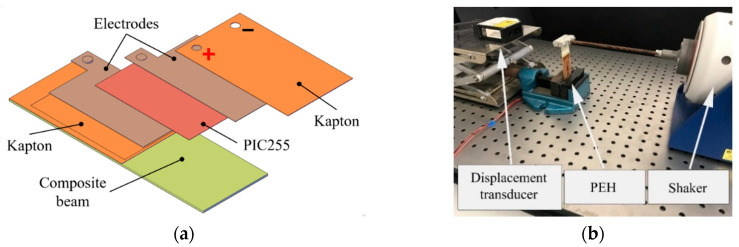
(**a**) Schematic drawing of the low-cost piezoelectric energy harvester (PEH) used in this work and (**b**) setup employed for its experimental characterization (the humidity generation chamber is not shown in this figure).

Tests were performed on a home-made cantilevered piezoelectric energy harvester (Figure 2a), which consisted of a bendable piezoelectric patch (PZT, Physik Instrumente GmbH, Karlsruhe Germany, mod. DuraAct P-876 A.12) glued onto a composite beam. The PZT was made of a modified lead zirconate titanate powder (PIC255), enclosed into a polymeric case with dimensions of 61 mm × 35 mm × 0.5 mm, which ensured a high resistance to cyclical loads [28]. It featured a piezoelectric material thickness of 200 µm, a d_31_ charge coefficient of −180 pC/N, a capacitance of 90 nF, a blocking force of 265 N, a minimum lateral constriction of 650 µm/m, and a bending radius of 20 mm. The maximum operating frequency was 1 × 10^4^ Hz, the fatigue limit was up to 1 × 10^9^ cycles, while the operating temperature ranged from −20 to +150 °C [14].

The cantilever beam was obtained by the manual layup of three layers of 0°/90° oriented fiberglass and epoxy resin with dimensions of 105 mm × 35 mm × 1 mm. This type of generator shows a symmetric structure during its deformation; therefore, the same quantity of charge is generated, although with the opposite sign along two following half-periods of the vibration.

In the experimental setup (Figure 2b), the PEH was locked at one end to a vise and to the stinger of an electrodynamic shaker (Tira GmbH, Schalkau, Germany, mod. S 51) at the other end. The shaker was driven by a power amplifier (Tira GmbH, Schalkau, Germany, mod. BAA 120) and a function generator (Agilent Technologies Inc., Santa Clara, CA, USA, mod. 33220 A). An oscilloscope (Tektronix Inc., Beaverton, OR, USA, mod. TDS 2014) equipped with a 10 MΩ probe was employed to measure the PEH output voltage as the electrical load is varied, while a laser displacement transducer (MicroEpsilon GmbH, Ortenburg, Germany, mod. ILD 2200-50) was used to measure beam deflection.

### 2.2. Effects of Vibration Frequency and Working Temperature

The PEH electro-mechanical behavior was investigated setting the displacement amplitude of the shaker stinger at 2 mm, that is, ±1 mm from the equilibrium position. The PEH output voltage across the applied electrical load was measured for different vibration frequencies and electrical resistance loads. Moreover, tests were also performed by varying the PEH temperature. Temperature tests were carried out by putting the PEH and associated electronics into a benchtop humidity generation chamber (Thunder Scientific Corp., Albuquerque, NM, USA, Model 2500) able to maintain relative humidity in the range of 10–95% with 0.5% uncertainty and a temperature up to 70 °C with 0.06 °C uncertainty. All the tests were carried out fixing RH to 45%.

The PEH power output *P_PZT_* was calculated as follows:

(1) $$P_{PZT}=\frac{v_{PZT}^{2}}{8R_{L}}$$

where *v_PZT_* is the PEH peak-to-peak output voltage and *R_L_* is the electric resistance load, downstream of the PEH.

A detailed analysis of the conversion efficiency of the PEH used in this work has been recently published elsewhere by some of the authors of the present work [29].

**Figure 3:**
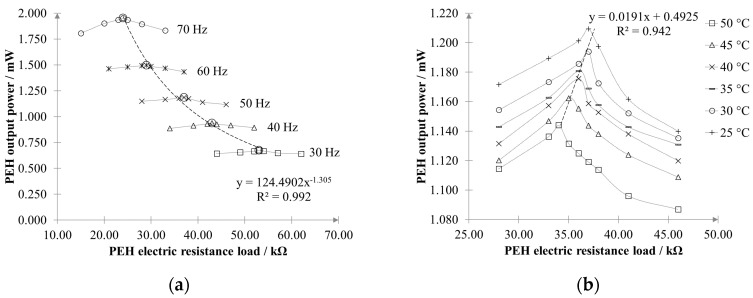
(**a**) Effect of the vibration frequency on PEH output power (at a fixed temperature of 25 °C) and (**b**) effect of the working temperature (while keeping the vibration frequency at 50 Hz).

Figure 3a shows the output power measured at fixed temperature (25 °C) as a function of the electrical load, for various working frequencies, while Figure 3b highlights the effects of varying the working temperature. The PEH output power increases with the vibration frequency *f_m_* and decreases with the operating temperature *θ_PZT_.* For given values of *f_m_* and *θ_PZT_*, an optimal load resistance *R_L___opt_ (f_m_, θ_PZT_)* exists that maximized the generated power. Such an optimal load varies almost hyperbolically with the vibration frequency and almost linearly with the working temperature.

## 3. Design of Energy Harvesting Single-Stage Converter

**Figure 4:**
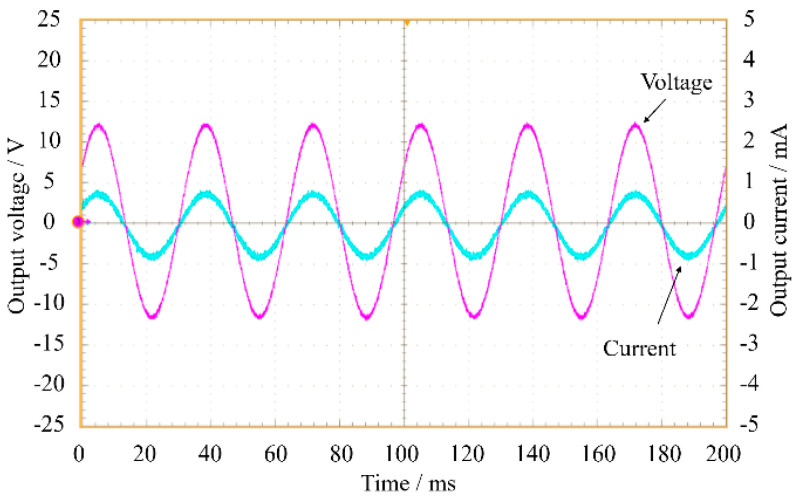
Typical PEH output voltage (in magenta) and output current (in cyan).

When cyclically loaded, piezoelectric harvesters generate an AC voltage whose amplitude is a function of the vibration frequency (Figure 4). In practical applications of PEHs, a power converter is usually connected downstream to achieve different but correlated tasks. Its first purpose is to rectify and stabilize the PEH output voltage, thus making the PEH itself suitable for supplying an electronic device or a battery. Consequently, it should be able to step up or down the input voltage with the goal to increase the energy efficiency, adapting the PEH impedance to a given electrical load.

**Figure 5:**
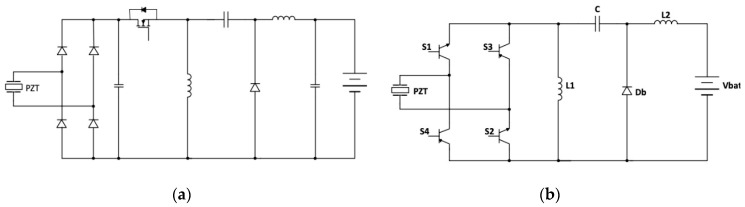
Power conversion circuits based on a diode rectifier and a standard Zeta converter: (**a**) two-stage converter and (**b**) proposed single-stage converter.

A further task comes directly from the results of the previous section and, specifically, from the need to match and track the resistance seen at the output of the PEH as it varies with time. In this way, the maximum output power would be guaranteed whatever the excitation source or the environmental temperature. In principle, a two-stage converter topology including a diode rectifier and a suitable DC–DC converter should be appropriate to achieve this goal. A possible solution is that shown in Figure 5a, where the front end is a full-bridge diode rectifier. This configuration is used to convert the AC input voltage into a DC voltage, which is stabilized by a suitable capacitor. The second stage is a Zeta converter, which regulates the output DC voltage. However, a circuit like this processes twice the output power, thus reducing the conversion efficiency. To overcome this drawback, a single-stage topology based on a Zeta converter was instead devised (Figure 5b). The proposed solution is a modification of the conventional single-switch Zeta converter [30]. Indeed, introducing an active rectifier, the original DC–DC converter is turned into an AC–DC converter.

As shown in Figure 5b, a full-bridge active rectifier replaces the single switch present in the basic Zeta converter. This has a multiple aftermath. First, the PEH output current is processed by two power devices instead of three, reducing the power losses. Second, the active rectifier can be designed to generate a very low voltage drop, avoiding the efficiency reduction caused, especially on low voltage circuits, by the diode fixed voltage drop. Finally, the active rectifier, unlike the diode rectifier that is a highly nonlinear load, can be driven to behave as a resistance. On the contrary, an active rectifier requires a suitable control system, which leads to extra costs and power self-consumption if compared with a diode rectifier. However, in the present case, this disadvantage is nearly removed, because the active rectifier replaces the switch of the conventional Zeta converter; thus, no additional control circuitries are needed. Moreover, the full-bridge driver circuit can be simplified by using a couple of NPN and PNP bipolar junction transistor (BJT) devices in a totem-pole configuration for each leg. Finally, an automatic input current shaping is achieved by operating the converter in discontinuous conduction mode (DCM). This makes no longer necessary a current control loop with related current sensing devices, enabling the use of a simple pulse-width modulation (PWM) strategy with constant duty cycle *δ* = *t_on_/T_s_* (where *t_on_* is the opening time and *T_s_* is the switching period) and switching frequency *f_s_*.

**Figure 6:**
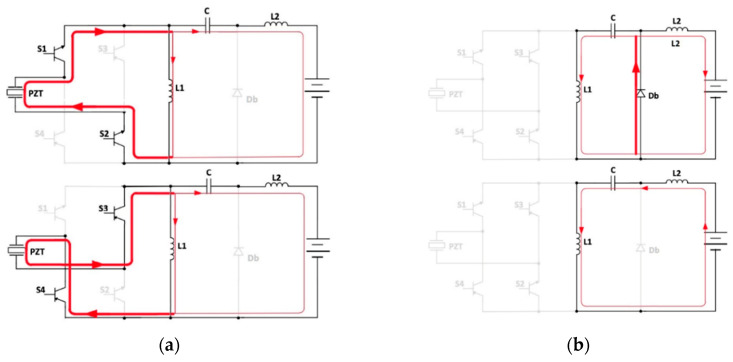
(**a**) Powering mode with *v_AC_*(*t*) *> 0* (up) and *v_AC_*(*t*) *< 0* (down); (**b**) free-wheeling mode (up), idle mode (down).

The DCM operation of the proposed converter is described in Figure 6. In powering mode, the input voltage *v_PZT_*(*t*) is applied to inductors *L*1 and *L*2, which are charged, as well as the coupling capacitor *C*. Specifically, in the positive half-period of *v_AC_*(*t*), the switches *S*3 and *S*4 are turned off and the other switches *S*1 and *S*2 are turned on, while during the second half-period of *v_AC_*(*t*), *S*1 and *S*2 are turned off and *S*3 and *S*4 are turned off. In free-wheeling mode, *S*1, *S*2, *S*3, and *S*4 are turned off, while the diode *Db* conducts. The energy stored in the previous step from the coupling capacitor *C* is transferred to *L*1, while *L*2 supplies the battery. When all the energy stored in *L*2 is transferred to the battery, the converter enters into the idle mode. The currents through the inductors become constant and the coupling capacitor *C* is charged to the battery voltage.

**Figure 7:**
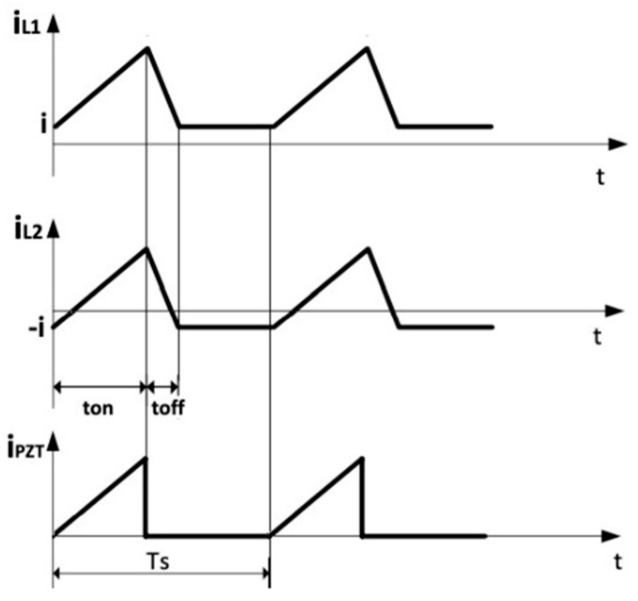
Inductors and input currents.

The resulting inductors and input currents are schematically shown in Figure 7.

The following mathematical dissertation is referred to [31]. Assuming a constant duty cycle operation on each half-cycle of the vibration, the average value on a switching period *T_s_* of the PZT output power *P_PZT_*(*t*) is given by the following:

(2) $$P_{PZT}\left(t\right)=\frac{v_{PZT}^{2}}{2\;f_{s}}\left(\frac{1}{L_{1}}+\frac{1}{L_{2}}\right)$$

The converter is seen by the PEH as a controllable resistance *R_L_(δ)*, given by the following:

(3) $$R_{L}\left(\delta \right)=\frac{2f_{s}L_{1}L_{2}}{\left(L_{1}+L_{2}\right)\delta^{2}}$$

In order to maximize the energy yield, the electrical resistance should be adapted to cope with variations in vibration frequency and temperature, as already highlighted in Figure 3a,b. The optimal duty cycle *δ_opt_* can be determined by setting *R_L_(δ) = R_L_opt_ (f_m_, θ_PZT_)*, as follows:

(4) $$\delta_{opt}=\sqrt{\frac{2f_{s}L_{1}L_{2}}{\left(L_{1}+L_{2}\right)R_{L_{opt}}\left(f_{m},\;\theta_{PZT}\right)}}$$

**Figure 8:**
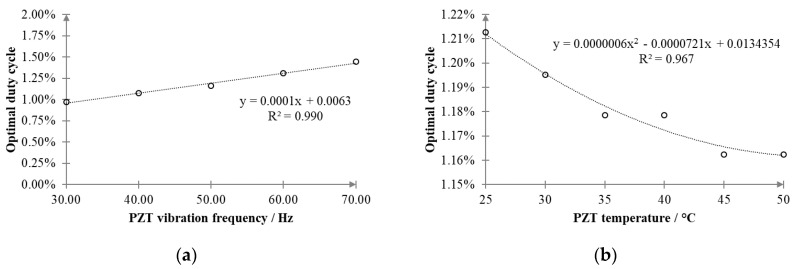
Effect of (**a**) the vibration frequency and (**b**) the temperature on the optimal duty cycle *δ_opt_*.

In principle, *R_L_opt_ (f_m_, θ_PZT_)* can be estimated by measuring the vibration frequency and the temperature from Figure 3a,b, as shown in Figure 8. However, this approach is quite impractical since it would require a preliminary full characterization of the PEH device.

**Figure 9:**
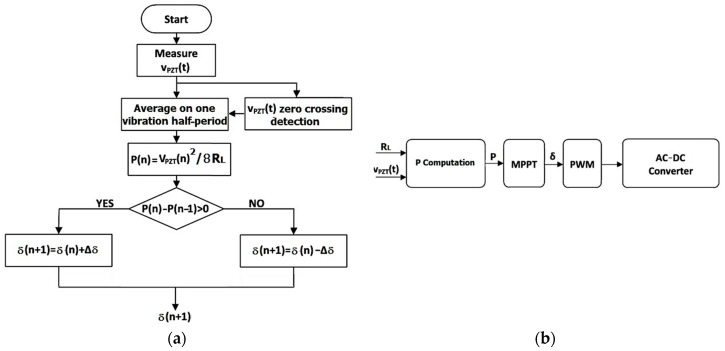
(**a**) Flowchart of the perturb and observe (P&O) maximum power point tracking (MPPT) algorithm and (**b**) converter control system with MPPT.

A more viable and general approach consists of adapting the duty cycle *δ* in order to force the PEH to always operate at its optimal working point. An MPPT algorithm, based on a perturb and observe (P&O) approach [32], was thus developed. The flow chart is displayed in Figure 9a. The adjustment step is one-half of the vibration period, being triggered by the detection of *v_PZT_*(*t*) zero crossings. In practice, the output voltage *v_PZT_*(*t*) is sampled, low-pass filtered, squared, and processed by an integrator, which is reset at each zero crossing. The result is divided by *R_L_*, which is obtained from Equation (2), to compute the average PEH output power over a half-period of the vibration. The obtained value is compared with that computed in the previous step. Based on the gradient of the output power, the duty cycle is then updated. The converter control system is very simple, being of the predictive type, as shown in Figure 9b.

Considering almost constant the PEH output voltage along the switching period *T_s_*, the small-signal based linearization of Equation (1), around a generic equilibrium point (*P_PZT_*_0_, *δ*_0_), gives the following:

(5) $$\Delta P_{PZT}=\frac{v_{PZT}^{2}\delta_{0}}{f_{s}}\left(\frac{1}{L_{1}}+\frac{1}{L_{2}}\right)G_{d}\left(s\right)\Delta \delta =F_{\delta P}\left(s\right)\Delta \delta$$

(6) $$G_{d}\left(s\right)=e^{-sT_{s}}\approx \frac{1-\frac{sT_{s}}{2}}{1+\frac{sT_{s}}{2}}$$

where Δ*P_PZT_* and Δ*δ* are power and duty cycle disturbances, respectively, while *G_d_*(*s*) deals with the delay caused by PWM.

According to the scheme of Figure 5a, a single-stage AC–DC converter based on a Zeta topology was then designed, featuring the technical specifications reported in Table 1.

The power consumption of the whole system mainly includes that from the microcontroller and from the switches. In detail, the microcontroller is set to operate in low consumption mode, i.e., using 10 µW. The average power consumption of the DC–DC converter is about 80 µW.

## 4. Validation Tests

**Figure 10:**
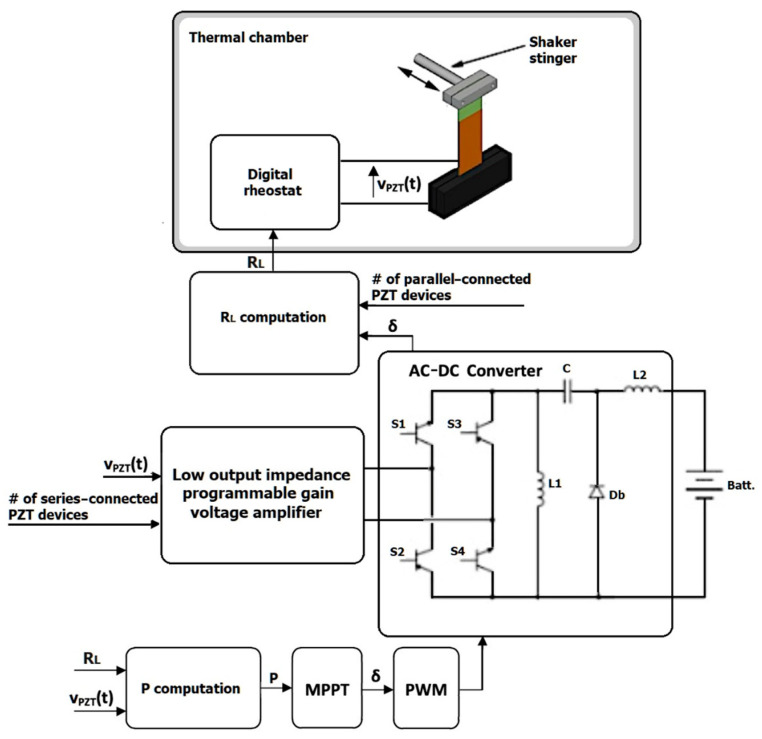
Schematic representation of the operating principle of the proposed active control strategy based on a single-stage DC–DC converter.

Figure 10 displays schematically the operating principle of the proposed active control strategy and single-stage DC–DC power conversion. Based on this scheme, a test bench was created to carry out experimental tests aimed at demonstrating the correct functioning of the proposed approach as either the vibration frequency or the working temperature was changed suddenly. In the figure, the PEH is coupled to a digital rheostat, while the converter is cascade-connected to a programmable gain voltage amplifier. This assembly enables an easy emulation of larger arrays made of multiple PEHs, either connected in parallel or in series [33,34]. The converter supplies the battery, which in turn powers the converter control system.

**Figure 11:**
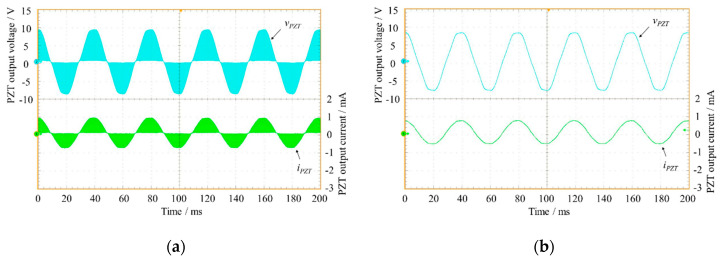
(**a**) Plots of output voltage *v_PZT_* (in cyan) and output current *i_PZT_* (in green) versus time and (**b**) filtered signals of the output voltage *v_PZT_* (in cyan) and of the output current *i_PZT_* (in green).

*v_PZT_*(*t*) and *i_PZT_*(*t*) signals are shown in Figure 11a. It can be highlighted that both presented a high-frequency component that had to be eliminated by low-pass filtering, resulting in the signals displayed in Figure 11b.

**Figure 12:**
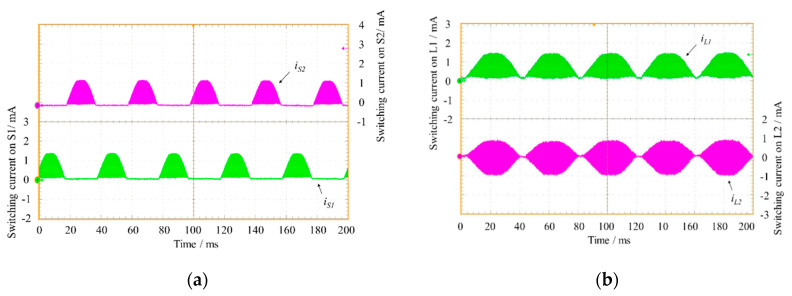
(**a**) Plots of switching currents *i_S_*_1_ (in green) and *i_S_*_2_ (in magenta); (**b**) plots of switching currents *i_L_*_1_ (in green) and *i_L_*_2_ (in magenta).

Figure 12a,b shows the transistor currents and the inductor currents according to the working condition explained in Figure 6, measured by means of Hall probes.

## 5. Discussion

PEHs in the form of cantilever beams are widely used for generating energy from low-frequency vibrating structures. To harvest the maximum energy from the piezoelectric element, impedance matching with the applied load is required. However, as was shown in Figure 3, the resistance load depends on both the vibration frequency and the working temperature; therefore, the variation of one of these two quantities leads to a shift of the optimal working point. In practical applications, it is unlikely that the excitation is pure harmonic or that the working temperature remains constant. Therefore, some way to track and match the optimal working point should be thought of if maximum power harvesting has to be achieved.

This paper faced this problem by exploiting an active control strategy based on a non-conventional AC–DC converter that integrates a DC–DC Zeta converter and a full-bridge active rectifier.

**Figure 13:**
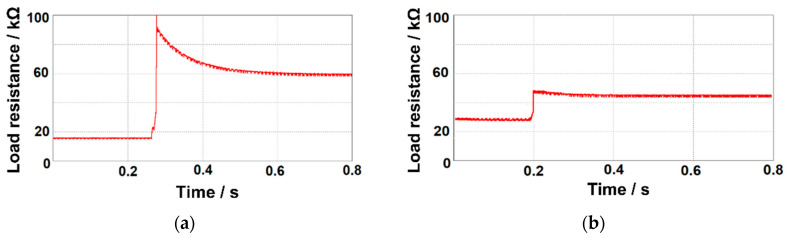
(**a**) Load resistance adaption (15–62 kΩ) coping with a vibration frequency variation from 30 to 70 Hz; (**b**) load resistance adaption (28–46 kΩ) coping with a PZT operating temperature variation from 25 to 50 °C.

To prove the effectiveness of the proposed approach, a prototype of the converter was created and tested with a cantilever PEH. Figure 13a,b shows the transient and steady-state response of the single-stage converter for a sudden change in the applied resistance load (which actually may occur if either the working frequency or temperature changes). Based on the results reported in Figure 3a,b, we induced a stepped change in the resistance load from 15 to 62 kΩ to emulate a change in the working frequency from 70 to 30 Hz, and from 28 to 46 kΩ, to emulate a change in the temperature from 25 to 50 °C. In both cases, the self-adaption capability of the single-stage AC–DC converter can be observed, which allows the PEH to always operate at its optimal working point, generating maximum power (see Figure 3a,b). Reducing the amplitude of the forced variation (i.e., the width of ΔR) only affects the time *t*_s_ needed by the converter to reach the steady-state condition, as it can be observed by comparing Figure 13a, in which ΔR is 47 kΩ and *t*_s_ is 0.38 s, and Figure 13b, in which ΔR equals 18 kΩ and *t*_s_ equals 0.15 s.

While the variability of the environmental temperature might be rather limited, this may not be the case as far the vibration frequency is concerned, as PEHs are usually called to work in a broadband excitation scenario, where multiple vibration frequencies coexist simultaneously. As shown in Figure 3, if we consider the PEH operating in optimal load matching at a temperature of 25 °C, without any adaptive compensation a variation of 30 Hz (from 30 to 60 Hz) of the vibrational frequency causes a −120% decrease in the generated power. On the contrary, at a fixed frequency of 50 Hz and optimum load, increasing the temperature from 25 to 50 °C produces only a −8% variation.

A specific consideration must be taken into account about the resonant frequency of the PEH since at resonance, as is well known, piezoelectric energy harvesters always generate the highest power [14,28]. In our tests, the tip of the cantilever was connected to the shaker (see Figure 2b) and it was forced to move with a fixed amplitude (it was not allowed to freely oscillate). Hence, it makes no sense to deal with resonance. It is noteworthy to highlight that this setup was deliberately chosen to study the intrinsic characteristics of the PEH rather than those of the assembly (which are function of the geometry of the system and of the actual constraint conditions). Moreover, the resonance condition is irrelevant for the performance of the proposed converter, as it adapts the impedance of the load whatever the working frequency. Letting the PEH work in resonance condition would only produce a higher generated power, without influencing the adaptative logic of the proposed power conversion scheme.

## 6. Conclusions

This paper presented an original approach, based on a single-stage AC–DC converter, aimed at tracking the optimal working point of piezoelectric energy harvesters. The proposed converter topology, based on an active control, consists of an AC–DC converter that integrates a DC–DC Zeta converter and a full-bridge active rectifier.

The study showed that the proposed strategy adapts the resistance seen by the PEH to the change in working conditions, allowing the piezoelectric harvester to always operate at its optimal working point, whatever the applied vibration frequency and/or the temperature, hence increasing the conversion efficiency.

To accomplish this, an active rectifier and a PWM strategy were used to control the designed converter, putting into evidence the fact that the optimal duty cycle is influenced by either the PEH excitation frequency or the working temperature. A dedicated algorithm of maximum power point tracking, based on a perturb and observe (P&O) approach, was developed. The validation tests highlighted the effectiveness of the proposed active controlled approach as the applied resistance load underwent a sudden change of up to 47 kΩ.

The proposed conversion topology introduces many advantages in the PEH electrical management, as shown in Figure 5b. The use of a full-bridge active rectifier allows to reduce the number of devices that process the current and the power losses. Moreover, a full-bridge active rectifier has a linear behavior, can be simplified using a couple of NPN and PNP BJT devices in a totem-pole configuration for each leg, and can replace the switch of conventional Zeta converters. For this latter reason, no additional control circuitries are needed. Total power consumption of the proposed converter is only 80 µW. This is much less than the minimum power generated by a single PEH, that is, 0.64 mW at 30 Hz as a resistance load of 62 kΩ is considered (see Figure 3a). Moreover, the power consumption of the converter does not depend on the number of PEHs that can be connected, in a parallel configuration, downstream to the converter. In this case, only the losses moderately increase because of the higher current. It is noteworthy to observe that a similar power consumption is expected if a conventional converter (without tracking capability) is used. Finally, since the converter operates in discontinuous conduction mode, it does not use a current control loop but it exploits pulse-width modulation, which is the core of the impedance tracking procedure. This guarantees a simpler and more accurate operation of the converter, since, unlike the voltage that easily reaches tens of V, the current supplied by a PEH is of the order of tens of µA.

## Figures and Tables

**Table 1:** Single-stage Zeta converter technical data. BJT, bipolar junction transistor.

PZT Max Peak Voltage	Battery Voltage	Switching Frequency	No. of Power Switches	Diode	Inductor L1	Inductor L2	Coupling Capacitor
20 V	5 V	50 kHz	4 BJT	1 N4008	100 µH	100 µH	100 µF
